# Quality of life in patients on chronic dialysis in South Africa: a comparative mixed methods study

**DOI:** 10.1186/s12882-016-0425-1

**Published:** 2017-01-05

**Authors:** Elliot K. Tannor, Elize Archer, Kenneth Kapembwa, Susan C. van Schalkwyk, M. Razeen Davids

**Affiliations:** 1 Division of Nephrology, Department of Medicine, Stellenbosch University and Tygerberg Hospital, Cape Town, South Africa; 2 Centre for Health Professions Education, Stellenbosch University, Cape Town, South Africa

## Abstract

**Background:**

The increasing prevalence of treated end-stage renal disease and low transplant rates in Africa leads to longer durations on dialysis. Dialysis should not only be aimed at prolonging lives but also improve quality of life (QOL). Using mixed methods, we investigated the QOL of patients on chronic haemodialysis (HD) and peritoneal dialysis (PD).

**Methods:**

We conducted a cross-sectional study at Tygerberg Hospital in Cape Town, South Africa. All the PD patients were being treated with continuous ambulatory peritoneal dialysis. The KDQOL-SF 1.3 questionnaire was used for the quantitative phase of the study. Thereafter, focus-group interviews were conducted by an experienced facilitator in groups of HD and PD patients. Electronic recordings were transcribed verbatim and analysed manually to identify emerging themes.

**Results:**

A total of 106 patients completed questionnaires and 36 of them participated in the focus group interviews. There was no difference between PD and HD patients in the overall KDQOL-SF scores. PD patients scored lower with regard to symptoms (*P* = 0.005), energy/fatigue (*P* = 0.025) and sleep (*P* = 0.023) but scored higher for work status (*P* = 0.005) and dialysis staff encouragement (*P* = 0.019) than those on HD. Symptoms and complications were verbalised more in the PD patients, with fear of peritonitis keeping some housebound. PD patients were more limited by their treatment modality which impacted on body image, sexual function and social interaction but there were less dietary and occupational limitations. Patients on each modality acknowledged the support received from family and dialysis staff but highlighted the lack of support from government. PD patients had little opportunity for interaction with one another and therefore enjoyed less support from fellow patients.

**Conclusions:**

PD patients experienced a heavier symptom burden and greater limitations related to their dialysis modality, especially with regards to social functioning. The mixed-methods approach helped to identify several issues affecting quality of life which are amenable to intervention.

**Electronic supplementary material:**

The online version of this article (doi:10.1186/s12882-016-0425-1) contains supplementary material, which is available to authorized users.

## Background

Chronic kidney disease (CKD) is an important public health problem which is increasing in terms of incidence and prevalence. The worldwide prevalence is 10–13% [1, 2] and similar estimates are reported for Africa (13.9%) [3]. End-stage renal disease (ESRD) is a serious complication of CKD and requires renal replacement therapy (RRT) in the form of haemodialysis (HD), peritoneal dialysis (PD) or transplantation as the mainstay of treatment.

Of the more than 1.8 million patients worldwide on dialysis, less than 5% are in Africa where access to RRT is dependent on very limited government support [4, 5]. The dialysis rates across Africa are less than 20 per million population (pmp) as compared to a global prevalence of 223 pmp. In Africa, 97% of dialysis patients are on HD [6]. South Africa has a higher proportion of patients on PD. According to recent registry data 71.8% of patients receiving RRT are on HD and 13.5% on PD [7].

RRT should not only prolong life but also sustain quality of life [5, 8]. Transplantation results in improved survival, lower costs and better quality of life [9–14] but transplant rates in Africa are very low, averaging only 4 pmp [15] and decreasing. In South Africa, the proportion of RRT patients with a functioning transplant has decreased from 55.5% in 1994 to 14.7% in 2014 [7, 16]. The low transplant rates and increasing numbers of patients requiring RRT result in longer durations on dialysis, emphasising the importance of assessing and optimizing the quality of life of our patients on chronic dialysis.

Health-related quality of life (HRQOL) has become increasingly important as an outcome measure of RRT. The traditional focus on the improvement of survival has recently shifted to include a much stronger emphasis on quality of life [8]. HRQOL represents the impact of the disease or its treatment on the subjective feelings of patients about their physical, mental, spiritual, emotional, social and functional wellbeing [17]. HRQOL deteriorates as kidney function worsens [18] and is an independent risk factor for mortality in dialysis patients [19]. The Kidney Disease Quality of Life (KDQOL) questionnaire [20] has been validated for use as a disease-specific measure of quality of life. It combines the SF-36 instrument with kidney disease specific items [21, 22].

Quality of life among dialysis patients has been shown to be lower as compared to pre-dialysis CKD patients [23], the general population [24] and other chronic diseases like congestive heart failure, diabetes, depression and even cancer [25].

In the large Dialysis Outcome and Practice Pattern study (DOPPS) in the United States [26] poor scores in the physical component of the HRQOL were associated with increased mortality and increased risk of future hospitalisation.

The quality of life of patients on PD has been reported to be better than for those on HD in some studies [27–30] but others, including a systematic review [14, 28, 31, 32], have reported no difference between the two treatment modalities. PD patients tended to have lower scores in the role-physical and bodily pain domains whereas HD patients had lower scores in the emotional component [33]. Low quality of life at the initiation of RRT is associated with increased hospitalisation and higher mortality, emphasizing the need for early interventions [34, 35].

Very few studies have been conducted comparing the quality of life of HD and PD patients in South Africa [28, 36, 37]. A study in the Western Cape, South Africa, reported low HRQOL in both dialysis modalities with no difference between HD and PD [28]. Another South African study [37] which included dialysis patients from both the private and public healthcare sectors also reported compromised quality of life in both modalities although HD patients were found to have increased vitality and better physical component scores.

There are no qualitative data available from these studies to provide a richer interpretation of the numbers reported. We therefore sought to study the quality of life of patients treated with PD and HD using a comparative mixed methods approach. Focus group interviews have the potential to generate rich data based on the synergy of group interaction and allowed us to ascertain our patients’ perspectives about factors affecting their quality of life.

## Methods

We conducted a cross-sectional study involving both qualitative and quantitative methods. Tygerberg Hospital is a public-sector 1384-bed teaching hospital in Cape Town, South Africa. The numbers of patients on RRT is limited to approximately 65 patients on HD and 55 patients on PD due to resource constraints. Only patients who are transplantable are accommodated and the renal replacement programme operates on a PD-first policy as there are limited numbers of haemodialysis slots available. A few patients are accommodated closer to their homes at satellite HD units in the nearby towns of Paarl, Worcester, Hermanus and Vredenburg.

All our HD patients receive three 4-hour, in-centre, treatments per week. High-flux dialysers and bicarbonate-based dialysate solutions are used. Continuous ambulatory peritoneal dialysis (CAPD) using a twin-bag system is the sole form of PD, with the majority of PD patients performing four 2-litre exchanges per day. None of our patients have access to a cycler for automated PD because of the substantially higher costs of this treatment option. Dialysis access is achieved via arterio-venous fistulae or tunnelled catheters for most HD patients. For the PD patients, Tenckoff catheters are inserted at the bedside, or in theatre by the surgical team in cases where there are likely to be intra-abdominal adhesions. All patients on chronic dialysis have access to erythropoietin and intravenous iron as required. Unfortunately, most patients present late and there are usually few opportunities for pre-dialysis counselling.

Dialysis patients were recruited from June 2015 and March 2016 if they had been stable on a treatment modality for more than three months. Participants with other chronic illnesses such as malignancy, chronic liver disease or stroke were excluded from the study. Patients admitted in the previous month for any acute illness were also excluded. Participants completed a validated, pre-tested, self-administered questionnaire, the KDQOL-SF 1.3 [20]. This instrument includes 43 items which are focused on the health-related concerns of patients with renal disease on dialysis (Table 2, first column) and 36 generic core items (Table 3, first column). Raw scores on the KDQOL-SF 1.3 are transformed to a score ranging from 0 to 100 with higher scores representing better perceived QOL.

Data for quantitative analysis were captured using REDCap [38] and analysed with Stata/SE 14 [39]. Data were summarized as means ± standard deviations, or medians and interquartile ranges if not normally distributed. The chi square and Fisher’s exact tests were used for categorical variables. Student’s *t* test was used to compare continuous variables, and the Wilcoxon sign rank test when not normally distributed. A *P*-value of <0.05 was considered statistically significant.

The qualitative phase of the study involved six hour-long focus group interviews [40], each conducted by an experienced registered nurse without any connection to the RRT programme. The interviews included three groups comprised of only HD patients and three groups of only PD patients. Each group included approximately six patients, with the participants in each group having been purposively sampled to ensure variation based on sex, race, language and dialysis vintage [41]. English and Afrikaans^1^ were the languages used; the facilitator was fluent in these languages. Prompts were used and leading questions avoided allowing participants the freedom to express themselves.

The interviews continued until data saturation was reached. Interviews were recorded electronically, transcribed verbatim and then analysed using thematic analysis with a view to identifying recurring patterns in the texts [42]. Accordingly, the transcripts were coded and subsequently thematic labels assigned manually [41].

## Results

### Quantitative phase

A total of 106 patients were recruited out of a total of 128 eligible dialysis patients. Eight patients could not be reached, 2 declined to participate and the remainder did not return their questionnaires, giving a response rate of 83.6%. Participants included 48 PD patients and 58 on HD.

Regarding the baseline characteristics (Table 1), the mean age in the PD group was lower (36.1 vs. 42.8 years, *P* = 0.001). Most of the participants were females and their ethnicity was mostly mixed ancestry (“Coloured”) [43], reflecting the population of the Western Cape. The median duration on RRT was 2.2 years for PD patients and 6.0 years for the HD patients (*P* < 0.001). Median duration on the current dialysis modality was 1.1 years and 3.2 years respectively (*P* = 0.003).

**Table 1:** Baseline characteristics of study participants

Variable	PD (*n* = 48)	HD (*n* = 58)	*P*
Age (years)	36.1 ± 10.7	42.8 ± 9.8	0.001
Female	27 (56.3)	41 (70.7)	0.123
Ethnicity			
Mixed ancestry	34 (70.8)	42 (72.4)	0.72
Black	9 (18.8)	12 (20.7)	
White	4 (8.3)	4 (6.9)	
Duration on RRT (years)	2.2 (0.6–4.1)	6.0 (3.4–10.9)	<0.001
Duration on modality (years)	1.1 (0.2–3.3)	3.2 (1.2–6.0)	0.003
Haemoglobin (g/dl)	8.8 ± 2.1	10.5 ± 1.9	0.001
Albumin (g/l)	35.0 ± 5.7	37.9 ± 3.9	0.004
Total calcium (mmol/l)	2.18 ± 0.3	2.19 ± 0.3	0.855
Phosphate (mmol/l)	2.0 ± 0.8	1.71 ± 0.8	0.044
PTH (pmol/l)	48.3 (25.4–130.7)	43.0 (17.8–95.6)	0.198
spKt/V		1.44 ± 0.28	

Data expressed as mean ± standard deviation, median (interquartile range) or as number (percentage)

*Abbreviations: RRT* renal replacement therapy, *spKt/V* single pool Kt/V

For the HD patients, dialysis access was achieved via arterio-venous fistulae in 25 patients (41.4%), tunnelled catheters in 28 (48.3%), temporary dialysis catheters in 4 and via arterio-venous grafts in 2 patients. Dialysis adequacy in the HD patients was good with a mean single pool Kt/V of 1.44 ± 0.29. Adequacy was not measured routinely in the patients on PD. The HD patients had higher mean haemoglobin and serum albumin concentrations, and lower serum phosphate concentrations (Table 1). While we do not have complete data on peritonitis, a retrospective review of the period of the study revealed that peritonitis rates were high, exceeding 1 episode per patient per year.

**Kidney-disease-targeted items** (Table 2). Overall, there was no difference between the groups. There were, however, differences in the symptoms, work status and sleep domains. PD patients scored lower with symptoms and sleep, and scored higher for work status and dialysis staff encouragement than those on HD.

**Table 2:** Comparison of the KDQOL-SF 1.3 scores of the kidney-disease-targeted items between PD and HD patients

Disease-targeted items	PD (*n* = 48)	HD (*n* = 58)	*P*
Symptoms	63.8 ± 19.5	73.6 ± 15.6	0.005
Effects of kidney disease	60.9 ± 25.9	63.5 ± 25.0	0.601
Burden of kidney disease	42.8 ± 32.8	47.9 ± 33.4	0.460
Work status	41.7 ± 42.9	19.3 ± 32.4	0.005
Cognitive function	72.0 ± 18.9	77.9 ± 21.0	0.135
Quality of social interaction	68.1 ± 20.3	71.0 ± 17.7	0.433
Sexual function	71.1 ± 19.0	65.9 ± 33.1	0.516
Sleep	55.5 ± 16.1	62.9 ± 16.9	0.023
Social support	79.5 ± 24.4	82.2 ± 23.5	0.569
Dialysis staff encouragement	93.5 ± 11.8	86.4 ± 17.4	0.019
Patient satisfaction	72.9 ± 23.2	63.5 ± 26.4	0.056
**Total**	**65.4 ± 13.7**	**65.1 ± 11.7**	**0.903**

Data expressed as mean ± standard deviation or median (interquartile range)

*Abbreviations: RRT* renal replacement therapy

For the **generic core SF-36 items** (Table 3), there was also no difference between the groups. The HD group, however, scored higher in the social functioning and energy/fatigue domains. Overall KDQOL-SF 36 1.3 scores were not different for the two modalities.

**Table 3:** Comparison of the generic core 36-item health survey of the KDQOL-SF36 1.3 questionnaire in PD and HD patients

SF-36 item health items	PD (*n* = 48)	HD (*n* = 58)	*P*
Physical function	55.5 ± 21.7	54.7 ± 19.4	0.829
Role physical	25.5 ± 34.0	33.8 ± 38.5	0.314
Pain	61.6 ± 29.9	60.2 ± 29.5	0.811
General health	47.9 ± 22.3	48.7 ± 21.9	0.850
Emotional wellbeing	62.7 ± 19.7	68.6 ± 17.9	0.112
Role-emotional	49.6 ± 45.0	41.4 ± 41.6	0.362
Social function	54.9 ± 26.4	67.5 ± 22.2	0.009
Energy and fatigue	45.0 ± 18.0	52.2 ± 14.4	0.025
Total	50.5 ± 18.2	53.5 ± 17.4	0.386
**Overall KDQOL-SF1.3 score**	**57.9 ± 14.3**	**59.3 ± 13.2**	***P*** **= 0.612**

Data expressed as mean ± standard deviation or median (interquartile range)

### Qualitative phase

#### Participants in the focus group discussions

A total of 17 participants (11 females) on PD took part in the focus group discussions. Their mean age was 39.4 years (range 21–64 years), with 2 black patients, 2 white patients and 13 who were of mixed ancestry. The median duration on any form of RRT was 3.0 years (range 0.3–23.0 years) and the median duration on PD was 1.6 years (range 0.3–8.7 years).

For the HD focus groups, there were 19 participants (11 females) with a mean age of 38.2 years (range 20–59 years). They included 7 black patients and 12 of mixed ancestry. All but one of the HD patients had previously been treated with PD. The median duration on RRT was 6.8 years (range 0.8–26.3 years) and the median duration on HD was 2.8 years (range 0.5–13.8 years). Three patients had had a previous kidney transplant.

The results of this phase of the study are discussed below under the various themes which emerged during the thematic analysis.

#### Symptoms and complications

Reported symptoms and complications included dialysis access infection, insomnia, itching, decreased libido and decreased sexual performance. With regards to dialysis access, peritonitis was noted as a common complication by the PD group while the HD patients did not verbalise any vascular access issues. In fact, fear of developing peritonitis kept some PD patients housebound, since they were worried that they would get infected if they did their exchanges outside their homes.

*“When you wake up in the morning, the stomach is like cramping …”* (PD, male)

*“It’s outside, going outside I have to worry about. The temperature I have to worry about, the weather, how cold, how wet, how windy, and everything.”* (PD, female)

PD patients found it difficult to sleep at night while the HD patients reported sleeping better than when they were on PD. Itching was verbalised as a cause of the insomnia by some patients on PD. There were no comments on itching in the HD group.

*“You can’t help it. You don’t want to scratch, but it’s itching.”* (PD, female)

*“In the beginning when I started on Haemo I could not sleep at night, but it got better and now it is gone.”* (HD, female)

Both groups described decreased desire for sex, and some of the male HD patients complained of erectile dysfunction.

*“I’m too weak for the girls. I can’t perform.”* (HD, male)

#### Limitations

Patients verbalised treatment-related limitations involving their diet, occupations, social interactions, body image and sexual life. The effect on body image was only expressed in the PD group, and mainly among female participants who said that both they and their partners did not find their abdomens with the visible peritoneal dialysis catheter a pleasant sight.

*“… you are not feeling like a person anymore. You don’t want to undress in front of him …”* (PD, female)

*“… It’s [the catheter] not something you always want to look at every day… It really, really limits you.”* (PD, female)

*“You cannot, the way you had sex before, it’s not the same anymore …if he starts touching me, then I think: Oh God, the ‘pipe’, you know.”* (PD, female)

Dietary restrictions were noted by both groups as one of the key components that affected their quality of life. HD patients seemed to be more aware of their restrictions than their PD counterparts. Both groups reported limiting their water intake by sucking on ice to quench their thirst.

*Just for me not to be able to drink as much as I love to drink, that’s the worst thing for me, really, because I love tea.”* (PD, female)

*“We must drink ice, not water, but crushed ice”* (HD, Female)

Patients on both modalities were limited socially. Some PD patients stayed at home to do their scheduled bag exchanges and those who did go out would rush home in time for their next exchange. Young PD patients found it difficult to start and maintain relationships due to their kidney disease and had difficulty explaining themselves to prospective partners. HD patients mentioned that they could not travel away from their dialysis centres to visit family, attend key events such as funerals or go on vacations to places where there were no HD facilities.

*“… you can’t go with them because they won’t come back in time for you to do your exchange. So I rather stay at home…”* (PD, female)

*“It does limit you. It does limit you to your house.”* (PD, female)

*“You can’t even go on holidays because the hospitals in Eastern Cape* 2 *, they don’t have the support.”* (HD, male)

Another major limitation that was raised related to their occupations. While PD patients are usually considered to be more independent and able to work, most found it difficult to get employment, or to do their exchanges at work. Those who had a job felt that they were not seen as very productive by their employers. They stated that their employers were told by the doctors that PD patients are fit to work, and therefore expected a normal work performance from them despite the fact that some were still adjusting to PD and were often feeling very tired. HD patients generally did not work due to the need for hospital-based dialysis sessions three times a week. A few who managed to work on the non-dialysis days encouraged others to do the same.

*“…. you don’t get a job if you are on PD. People don’t want to employ us….”* (PD, male)

*“they don’t accommodate us as kidney patients in the workplace… sometimes it gets to the point where I just feel I’m really not doing PD today, it’s too much.”* (PD, female)

*“… it’s better when you are working. Now we can’t work.”* (HD, female)

#### Support

Patients found support from family, staff and fellow patients helpful in coping with their disease and maintaining their quality of life. Patients in both groups acknowledged that they were privileged to have good family support. Some HD patients, however, did not find the family supportive when it came to discussing issues around kidney donation. They blamed ignorance about kidney disease and inadequate education on organ transplantation as the main reasons why family members refused to be organ donors. Furthermore, they were of the opinion that the government did not support kidney patients as well as they supported patients with infectious diseases like HIV/AIDS and TB.

*“My family has been supportive since day one. ….my whole family started eating healthy.”* (PD, female)

*“…because now you want this kidney, they just turn their backs to you … the friends are running away.”* (HD, female)

*“Everyone has become obsessed with HIV. We know it is on everyone’s tongues. But nobody asked about kidney failure.”* (HD, female)

PD patients did not get much support from fellow patients since they usually had little interaction and did not know each other. Dialysis exchanges were done at home and they visited the hospital infrequently. They reported finding the focus group discussions very helpful and appreciated the opportunity to share experiences and ideas with each other. The following example illustrates this type of interaction.

*“Do you have those symptoms, the itchiness?”* (PD female)

A colleague answers: *“The itchiness, yes. But it’s not the pills. It’s the build-up of urea.”* (PD male)

HD patients, on the other hand, visited the hospital three times a week, often sharing the same hospital transport, and enjoyed a lot of support from fellow patients.

*“I was weak when I came here, and the support I got from the patients from the transport… I get strong.”* (HD, female)

*“…We speak the same language and encourage each other. This is important to us as patients and our lives.”* (HD, female)

The support received from the Renal Unit staff was described as good by patients on both modalities. This helped them to cope with their disease. Some PD patients complained that the staff were unhappy when they came in after hours with complications.

*“…they are professional and all, but they are like family.”* (HD, female)

*“They’re never miserable, they’re never moody, and even if they have issues at home they don’t bring it here.”* (PD, female)

## Discussion

The use of focus group interviews allowed us to explore patients’ perspectives and provided a rich source of qualitative data to illuminate the scores obtained from the questionnaires. We found no difference in the overall quality of life between HD and PD patients as measured by KDQOL 1.3 scoring. This finding is similar to that reported by other studies, including two conducted in South Africa [14, 28, 31, 32, 37]. Our overall QOL scores were somewhat lower than those reported from another public sector hospital in Cape Town [28] and comparable to a South African study which included patients from both private and public healthcare sectors [37].

Regarding the domains within the KDQOL 1.3 questionnaire, we found differences in the scores for specific domains between patients on the two dialysis modalities. PD patients had lower scores for symptoms, energy/fatigue, sleep and social function but better scores for work status and dialysis staff encouragement. The data which emerged from the focus interviews have shed more light on some of these findings.

The patients on PD verbalised more symptoms related to the ESRD and the complications of their dialysis modality. Peritonitis and catheter exit site infection was a common concern while access infections were not mentioned by the HD patients at all. Pruritus was a notable symptom which affected sleep in the PD patients but was not mentioned by the HD patients. Pruritus is common in dialysis patients and is associated with poor sleep, poor QOL and increased mortality [44]. Sexual dysfunction, including decreased libido and erectile dysfunction, were reported by patients on both modalities. The presence of the Tenckhoff catheter protruding from the abdomen negatively affected the body image and sexual function of the PD patients. HD patients reported symptoms suggestive of intradialytic hypotension and orthostatic hypotension after dialysis. These symptoms are also predictive of increased mortality [45]. Longer dialysis vintage may be associated with better QOL [46]. Our PD patients had a lower vintage and their symptom burden may reflect the initial difficulties in adjusting to life on dialysis, lower haemoglobin and albumin concentrations, as well as the occurrence of complications such as peritonitis and catheter exit site infection.

The lower social function score in the PD group was contrary to our expectation that PD patients would be more independent and therefore more likely to enjoy an active social life. This surprising finding was clarified in the focus group interviews where it became clear that the fear of developing peritonitis caused many patients to sacrifice social interactions to be able do their PD exchanges at home and at the specified times. Some felt that their home was the only safe place to do their exchanges. HD patients felt that their social interactions were limited by the need to remain close to their dialysis centres.

PD patients had better work status scores but nevertheless reported difficulties in finding employment and difficulties in doing bag exchanges while at work. They perceived their doctors and nurses as having unrealistic expectations of their ability to find and maintain employment. The treatment modality was considered to provide flexibility of lifestyle and leave them relatively independent but, in reality, this was mostly not the case. Their high symptom burden and the lack of support by employers in terms of providing time and space to do their bag exchanges made working very difficult. In contrast, the HD patients were generally understood to be unable to work due to the many hours per week spent on dialysis and travelling between their homes and treatment centres. Their doctors were more likely to support their applications for government social grants (“disability grants”). This discrepancy in the access to social grants was a major concern for the PD patients.

The dialysis staff encouragement domain had the highest scores for both groups of patients, although PD patients had the higher scores. Dialysis staff were described as being like family and this support helped improve their quality of life. The support of their families, fellow patients and also government support was verbalised as being extremely important. Social support exerts a strong independent influence on subjective and objective QOL [47]. PD patients called for the establishment of a patient support group and better financial support.

Reid et al. [48] conducted a systematic review to synthesize the experiences of patients on haemodialysis and identified four broad themes which emerged from the included studies: a new dialysis-dependent self, a restricted life, regaining control, and relationships with health professionals. Our findings can be mapped to all of these themes which could be used as a framework to consider interventions for improving the experiences of patients on chronic dialysis. Of note is that in our study relatively little data emerged on regaining control, possibly indicating an area where we should focus our own interventions.

The findings of the study have caused us to review several aspects of our management of patients on chronic dialysis. In view of our strict PD-first policy, it is especially important that we do more for our PD patients to optimise their dialysis, facilitate their social functioning and improve the support that they receive. This includes educating them that doing occasional exchanges early or late when they have important social events will not cause long-term problems. It also includes more readily motivating for government grants for those who are not well enough to work or cannot find work, having our social worker increase her efforts to enlist the support of employers and arranging regular meetings of support groups which include both HD and PD patients.

We have recognised that the increased symptom burden and the sub-optimal laboratory data of our PD patients may be due, at least in part, to chronic under-dialysis and high rates of peritonitis. This has prompted us to start a quality improvement project which will be focused on reducing peritonitis rates, monitoring the adequacy of dialysis and improving anaemia management. Expedited transplantation or a switch to haemodialysis will be considered more readily for patients who are clearly not thriving on PD.

Our study has several limitations. It is a single-centre, cross-sectional study with relatively small numbers of patients. The experiences of our patients may not be the same as those of patients managed in well-resourced settings where there is broad access to RRT and where patient choice plays an important role in determining the dialysis modality. The findings we have reported may be especially relevant to RRT programmes in resource-limited settings where a PD-first policy is being followed and where the availability of haemodialysis is limited. Another potential limitation relates to the selection of our study participants. The patients in the focus groups were not selected randomly, but purposively so as to ensure the inclusion of a variety of participants, and opinions, in terms of sex, ethnicity, language and dialysis vintage. Having both males and females in a focus group, as was the case in this study, could potentially inhibit discussion about issues such as sexual dysfunction. However, our facilitator was struck by the open discussion around sexual function and did not get the impression that the participants were inhibited at all.

## Conclusions

Our study has found that patients on PD were more symptomatic and experienced more treatment-related limitations than those on HD. In seeking to study what influences the quality of life of our dialysis patients we need to look beyond just the clinical issues and understand their real contexts. By applying qualitative research methods, clinicians may gain a deeper and more complete understanding of their patients’ experiences and help to identify issues affecting quality of life which may be amenable to intervention.

## Additional file

Additional file 1: Prompts for the focus group interviews. (DOCX 11 kb)

## Footnotes

1 Afrikaans is one of South Africa’s 11 official languages and the most spoken language in the province where the study was conducted.

2 The Eastern Cape is an under-resourced province in South Africa, especially as concerns dialysis and transplantation services.
