# Supplementary material for: Quality of life in patients on chronic dialysis in South Africa: a comparative mixed methods study
Source: BMC Nephrol. 2017 Jan 5;18:4. doi: 10.1186/s12882-016-0425-1 (PMC5217650; doi:10.1186/s12882-016-0425-1)
Supplement: Additional file 1: — Prompts for the focus group interviews. (DOCX 11 kb) [file 12882_2016_425_MOESM1_ESM.docx]

**Prompts for the focus interviews**

1. Begin with a general “ice-breaker” question like: how long have you been part of the specific group of patients - either PD or HD?

2. Ask about the patients’ general health while on PD or HD.

3. Effects of their kidney disease on their emotions, social and economic lives.

4. Their common symptoms and effect on their quality of life.

5. What their preferred treatment modalities would be and why.

6. Satisfaction with care received while on HD and PD.

7. What do they want changed, improved or abolished.
